# The role of trust and hope in antipsychotic medication reviews between GPs and service users a realist review

**DOI:** 10.1186/s12888-021-03355-3

**Published:** 2021-08-04

**Authors:** L. M. Grünwald, C. Duddy, R. Byng, N. Crellin, J. Moncrieff

**Affiliations:** 1 Division of Psychiatry, University College London, 149 Tottenham Court Rd, Bloomsbury, London, W1T 7NF UK; 2 Comprehensive Clinical Trials Unit, 90 High Holborn, London, WC1V 6LJ UK; 3 Nuffield Department of Primary Care Health Sciences, University of Oxford Radcliffe Observatory Quarter, Woodstock Road, Oxford, OX2 6GG UK; 4 Peninsula Medical School, University of Plymouth, Drake Circus, Plymouth, Devon, PL4 8AA UK; 5 Nuffield Trust, 59 New Cavendish Street, London, W1G 7LP UK; 6 Research and Development Department, North East London Foundation Trust, Maggie Lilley Suite, Goodmayes Hospital, Barley Lane, Ilford, Essex, IG3 8XJ UK

**Keywords:** Primary care, General practice, Antipsychotic medication, Medication review, Severe mental illness (SMI), Schizophrenia, Psychosis, Stigma, Trust, Shared decision making (SDM)

## Abstract

**Background:**

Increasing number of service users diagnosed with schizophrenia and psychosis are being discharged from specialist secondary care services to primary care, many of whom are prescribed long-term antipsychotics. It is unclear if General Practitioners (GPs) have the confidence and experience to appropriately review and adjust doses of antipsychotic medication without secondary care support.

**Aim:**

To explore barriers and facilitators of conducting antipsychotic medication reviews in primary care for individuals with no specialist mental health input.

**Design & setting:**

Realist review in general practice settings.

**Method:**

A realist review has been conducted to synthesise evidence on antipsychotic medication reviews conducted in primary care with service users diagnosed with schizophrenia or psychosis. Following initial scoping searches and discussions with stakeholders, a systematic search and iterative secondary searches were conducted. Articles were systematically screened and analysed to develop a realist programme theory explaining the contexts (C) and mechanisms (M) which facilitate or prevent antipsychotic medication reviews (O) in primary care settings, and the potential outcomes of medication reviews.

**Results:**

Meaningful Antipsychotic medication reviews may not occur for individuals with only primary care medical input. Several, often mutually reinforcing, mechanisms have been identified as potential barriers to conducting such reviews, including low expectations of recovery for people with severe mental illness, a perceived lack of capability to understand and participate in medication reviews, linked with a lack of information shared in appointments between GPs and Service Users, perceived risk and uncertainty regarding antipsychotic medication and illness trajectory.

**Conclusions:**

The review identified reciprocal and reinforcing stereotypes affecting both GPs and service users. Possible mechanisms to counteract these barriers are discussed, including realistic expectations of medication, and the need for increased information sharing and trust between GPs and service users.

**Supplementary Information:**

The online version contains supplementary material available at 10.1186/s12888-021-03355-3.

## Introduction

People with a diagnosis of schizophrenia or psychosis are often prescribed long term antipsychotic medication and treated in specialist secondary care services. However, people are now increasingly discharged to primary care and thus no longer have access to specialised care. In the UK, it is estimated that approximately 30% of people diagnosed with SMI are under primary care only [1–3]. Recently, this may have increased further, with some NHS trusts advising community mental health teams to discharge as much as 20% of their caseload to primary care due to the Covid − 19 pandemic.

There is significant literature on shared care agreements between secondary and primary care services to provide treatment for people with schizophrenia or psychosis, however little focuses on those people who are discharged from secondary care. This paper specifically aims to investigate the medication reviews of those people diagnosed with schizophrenia and/or psychosis, who no longer receive support from secondary care services.

In the UK, the Quality and Outcomes Framework requires a yearly health review for those on the SMI register, which should include the review of antipsychotic medication. There is however a paucity of research exploring the actual processes and content of antipsychotic medication reviews in primary care. Guidance also recommends that patients should be “on the lowest possible dose” [4] to avoid adverse reactions, however it is also unclear how this is to be achieved in primary care. This research is important, as studies have indicated that GPs feel that anti-psychotic medication prescribing is beyond their remit, and studies report a lack of knowledge and confidence in prescribing this medication [5]. Audits have also highlighted issues with current antipsychotic prescribing, including polypharmacy, dosages above BNF limits and off-label prescriptions [6, 7].

Antipsychotic medication deserves specific attention, as it is the main treatment for people with psychosis or schizophrenia. It is effective in reducing psychotic symptoms and reducing the risk of relapse, but it is also associated with serious side effects, including sedation and reduced motivation [8], sudden cardiac death [9], cardiovascular disease [10] and possibly decreased brain volume [11, 12] and cognitive impairment [13]. Although long-term prescribing has been the norm for decades, there is also a good rationale to suggest that not everyone requires long-term treatment or derives more benefit from it than harm [14, 15].

Therefore, antipsychotic medication should be reviewed regularly and appropriately to ensure that it is prescribed appropriately. This may be particularly pertinent for primary care only patients, who are on average older, on more medication and have been diagnosed for longer than service users (SU) still under secondary care [2, 3]. Reducing antipsychotic medication should be one of the options considered, since this has the potential to lower the risk of cardiovascular events and to reduce immediate side effects and thus improve quality of life. Such decisions need to be balanced against risks of relapse and suicide. It is unclear as to how primary care clinicians should be best supported in such important decisions. The extent to which GPs feel they can have discussions about antipsychotic medication in this population is unclear, especially given the lack of guidance available on what constitutes an appropriate medication review [7, 16].

### Aim

The aim of this study is to explore what works, for whom, how, in what respects, to what extent and in which contexts, for medication reviews conducted in primary care for SUs diagnosed with SMI. Specifically, we explored potential barriers and facilitators to conducting comprehensive medication reviews from a GP and SU perspective, for those SU who have been discharged from secondary care services.

## Method

Realist methodology is a theory driven approach, used to assess complex evidence relating to the implementation of policy, programmes, services and interventions [17]. It is concerned with understanding context in relation to underlying mechanisms of action and aims to address the key question: what works, for whom, under what circumstances and how? (as opposed to simply, “does it work?”) [17]. For this review, the stages outlined by Pawson (2006) were followed, as well as the Realist And Meta-narrative Evidence Syntheses: Evolving Standards (RAMESES) reporting and quality standards [18, 19].

A realist review was conducted to permit exploration of the underlying factors which might influence medication reviews in primary care and the development of a testable, programme theory, which could guide further research in this under researched area [17, 20]. This synthesis produced realist ‘context-mechanism-outcome configurations’ (CMOCs, see Table 1 for Glossary) that describe and explain the contexts and mechanisms likely to generate important outcomes relating to antipsychotic medication reviews [17], including ways to improve prescribing and remove some of the barriers relating to stigma and stereotypes in clinician and SU interactions, for those service users who have been discharged from secondary care.;

**Table 1:** Glossary

Term	Definition
Attribution Theory	a theory which supposes that people attempt to understand the behaviour of others by attributing feelings, beliefs, and intentions to them [23].
Context (C)	Elements outside the parameters of the formal programme architecture, that have causal impact, e.g. norms and values, economic conditions, participant characteristics
Context Mechanism Outcome Configuration (CMOC)	Configuration of the contexts, which trigger a mechanism, which results in an outcome.
Diagnostic Overshadowing	Misattribution of person’s symptoms as part of their mental health diagnosis rather than a co-morbid physical health issue. This can lead to incorrect diagnosis and/or delayed treatment.
Mechanism (M)	M is the underpinning generative force that leads to outcomes, triggerered by Context
Medication Review	In this review, a discussion between GP and SU to discuss the appropriateness and acceptability of their antipsychotic medication, including side effects, efficacy with regards to mental health and physical health.
Outcome (O)	Any result of a programme or study, can be intended or unintended, expected or unexpected
Programme Theory (PT)	A hypothesised theory made up of CMOCs, developed throughout the review (initial programme theory to refined programme theory)
Realist And MEta-narrative Evidence Syntheses: Evolving Standards (RAMESES)	Quality and publication standards and training materials for realist research approaches, funded by the National Institute of Health Research (NIHR) Health Services and Delivery Research Programmes.
Substantive Theory	A higher-level conceptual theory that is not directly about the programme, but introduces a concept(s) that increases the explanatory power of the programme theory

**Fig. 1:**
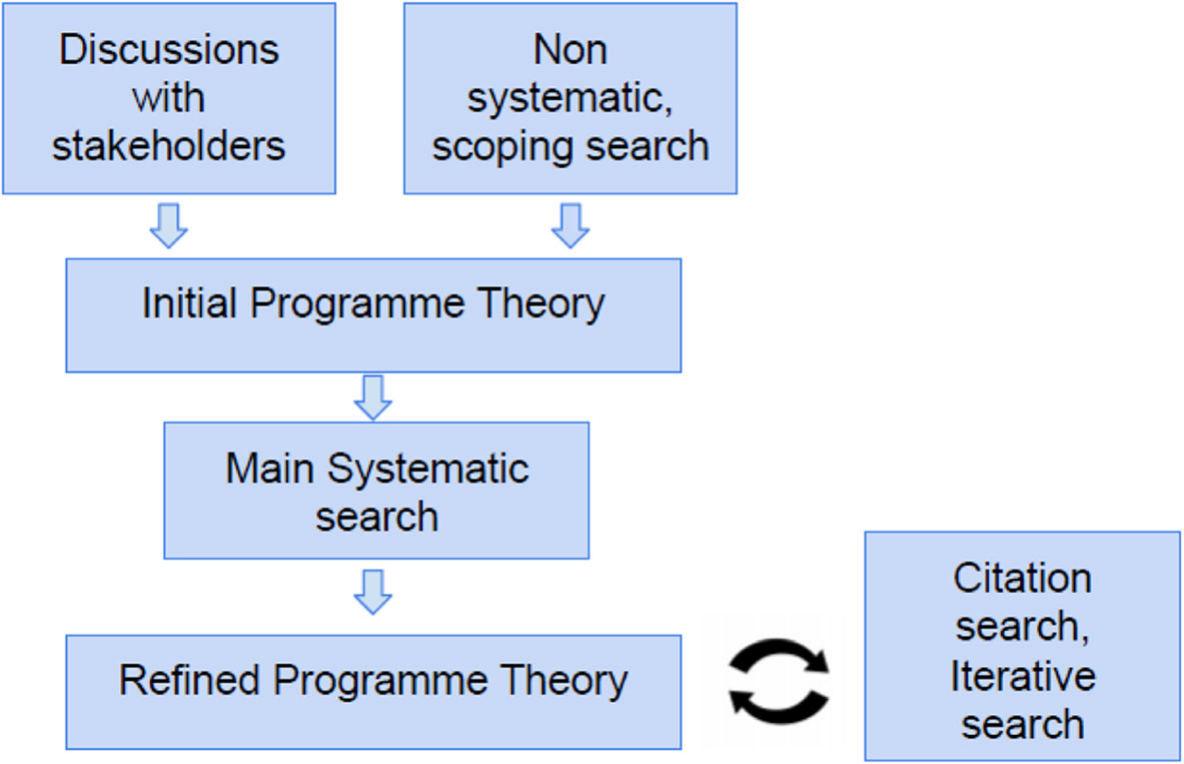
Data sourcing and PT development- Flow Chart

The full protocol is available elsewhere (Prospero CRD42018107573). The stages of the review process were as follows: 1) focusing the scope, 2) searching for evidence, 3) document selection, 4) data extraction and 5) data synthesis [17], as illustrated in Fig. 1. At each Step, the review processes were informed by discussions with wider stakeholders: Initially, the authors met with a member of the local CCG and their primary care mental health lead on three occasions, and held several conversations throughout the review process with GPs, a GP liaison psychiatrist and secondary care psychiatrists. This review is part of a wider NIHR funded programme – the RADAR trial on antipsychotic medication reduction. LG, NC and JM are also affiliated with the RADAR trial [21] and members of he trial’s LEAP (Lived Experience Advisory Panel), which included mental health service users and carers, were involved in discussing the initial aims of the study and reviewing theories on two occasions.

Following these initial discussions, the topic area for this review was narrowed down based on available evidence, and discussions with the project team, in line with RAMESES Quality Standard 3 [19]. The review focus is on GP and primary care only patients’ medication reviews, leaving out, for example, consideration of the factors affecting SUs making or attending appointments, and communication with, or prescribing done previously in secondary care settings, as these were considered to be out of scope. However, some documents that included data relating to GP views of secondary care were still included, as early discussions indicated that these views may play a role in the conduct of medication reviews for primary care only service users.

For step 2, a systematic search of 11 databases was conducted to identify studies containing relevant data for analysis. Two librarians aided the first author in refining the search terms and developing the search strategy. Medline (via HDAS), EMBASE (via HDAS), The Cochrane Library, CINAHL (via HDAS), PsycINFO (via HDAS), PsycEXTRA, the Web of Science Core Collection, Scopus, IBSS, OpenGrey and PubMed (via HDAS) were searched in August 2018. Search terms included variations of terms for “antipsychotic medication” and “primary care”. Initial scoping searches indicated a paucity of papers specifically discussing antipsychotic medication management in primary care settings, so the search strategy for the main search was designed to maximise sensitivity, and reduce the risk of missing data related to any potential contexts or mechanisms (“Big Bang Approach” [22];. Search results and results from the initial scoping searches were screened for eligibility based on the criteria in Table 2. Citation searches were run in April 2019.

**Table 2:** Inclusion and exclusion criteria

Inclusion criteria	Exclusion criteria
Adults (age 18 and above)	Service users currently under section (Mental Health Act, Forensic, Community Treatment Order) or currently in crisis or studies discussing Crisis services (Home Treatment Team etc)
Diagnosis of Psychosis, schizophrenia, psychosis like symptoms (SMI)	Animal studies
Medication reviews, care and treatment of service users diagnosed with SMI	Physical health reviews only, which do not include factors around treating SU or have medication reviews alongside
Published after 1954 (year the first antipsychotic was introduced)	Studies discussing prescription of non-antipsychotic medications
Published in English language	Studies from low- and middle-income countries
All study methodologies	Studies discussing the prevalence, and treatment of side effects by adding other (non-antipsychotic) medications
Prescription of antipsychotic medication in primary care	Studies discussing the prevalence or validity of a diagnosis of severe mental illness
	Off – label prescribing
	Excluded later:
	• Studies investigating bipolar disorder
	• Clozapine

The synthesis of data extracted from documents identified by the main and citation searches suggested that stereotypes and stigma were important mechanisms, therefore one further non-exhaustive, purposive search was conducted in August 2019 using relevant search terms to identify additional evidence related to these mechanisms The full search strategies are available in additional file 1).

For step 3 (document selection), all papers were screened by LG, first by title and abstract, and then in full text, with a 10% random subsample screened in duplicate by CD.

All included documents and data were critically appraised and assessed for rigour and relevance [20] in a 2-step process, adapted from Jagosh et al. (2011) and Francis -Graham et al. (2019) [24, 25]. Please see Additional Files for Template.

1. Overall quality appraisal (Additional Files 2) assessing the extent to which each document contributed *relevant* data (relating to contexts, mechanisms or outcomes) and assessing each document for *rigour* overall (using the Mixed Methods Appraisal Tool [26] or CASP Systematic Reviews [27], where possible).
2. Individual CMOC appraisal (Additional File 3) assessing the set of documents that contributed data to each CMOC in relation to their *relevance* to each CMOC (as each document contributed to CMOCs to a different extent) and *rigour*, i.e. the quality of their contribution to the CMOC (as each included document may have contributed a different type of data).

The results of the extraction and quality appraisal process are detailed in full in the additional files, to provide transparency with regards to each CMOCs’ evidence base.

For steps 4 and 5 (data extraction and data synthesis), included documents were read in full and coded by LG, with a 10% random subsample coded in duplicate by CD. All included papers from full text screening were added to NVivo (version 12.6.; qualitative data analysis software) and were initially coded into descriptive categories, which could shed light on potential contexts, mechanisms or outcomes.

Data codes were iteratively refined, and explanatory CMOCs were developed on the basis of the coded data. This included several rounds of reading all available documents and highlighting individual C, M, and O connections. As the review progressed, it became apparent that the literature was largely written either from a GP or from a SU perspective and that the key outcome is whether medication reviews happen or not, rather than the content of medication reviews themselves. Therefore, the data was grouped into “GP perspective” and “SU perspective” based on data available. The process also included extracting data on potential barriers and facilitators, as well as potential alternative outcomes to the medication review, and data highlighting important contexts. Barriers and facilitators were theorised based on the data available. This included considering contexts or mechanisms, based on data available, which would counter act the identified barriers. Further searching was used to identify additional documents that contained data used to refine the developing theories, as described above in step 2. Once all data was were coded, individual C,M and Os were written on notes and arranged to allow researchers to develop the final Programme Theory (PT).

The process of refining the programme theory involved retroductive reasoning, based on appraising and juxtaposing data extracted from the included documents. The developing analysis and explanatory CMOCs were discussed with stakeholders and refined further on the basis of their feedback. The CMOCs together allowed formation of an overall explanatory PT which was tested and refined throughout the review through these processes of data triangulation. This included sharing the PT with stakeholders for feedback and further refinement.

This overall Programme Theory describes identified barriers and facilitators to discussions about antipsychotic medication during GP appointments. Once the final PT was developed, another round of data extraction was completed to minimise chances of missing data,

## Results

**Fig. 2:**
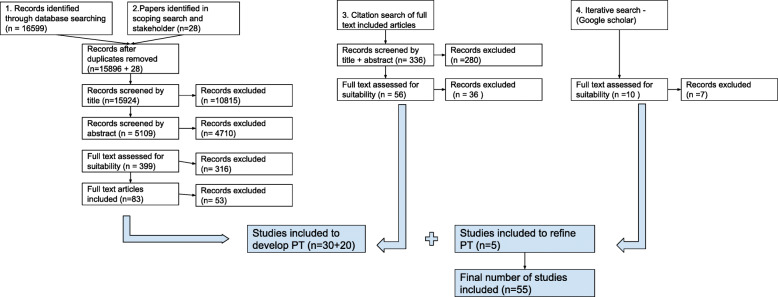
Literature Search

A total of 55 papers were included in this review (for details, please see Fig. 2 for the literature search results, Table 3 for a summary of papers identified. A full list of included papers is available in Additional File 4). No studies or guidelines directly exploring the needs of GPs or primary care only SU with respect to antipsychotic medication were found, despite a comprehensive search, illustrating the lack of research in this area. In particular, little evidence was found in relation to facilitators of antipsychotic medication reviews. As there is lack of research in this area, direct evidence on our population and setting of interest was unavailable to us. We therefore adopted a strategy of ‘borrowing’ data from comparable contexts, as is common in realist reviews, supported by feedback from our stakeholders. We are hypothesising that some mechanisms which occur in secondary care may also occur in primary care settings. For example, the experience of service users may be the same: some SUs describe their experience of medication reviews and/or queries with their GP, but since they were recruited through secondary care, the locus of care noted in Table 3 is still secondary care.

**Table 3:** Search results

Source identification	30 articles main search, 20 citation search, 5 iterative searches
**Design**	34 empirical studies (largely questionnaires and qualitative interviews), 1 systematic review, 16 non-systematic literature reviews, 4 other
**Topic**	27 care and treatment of people diagnosed with SMI (of which 10 guidance for GPs, 7 GP surveys on treatment of people diagnosed with SMI), 21 experience of taking antipsychotics from SU perspective, 7 stigma and Shared Decision Making
**Country**	31 = UK, 10 = USA, 7 = Australia, 3 = Canada, 1 = Ireland, 1 = Italy, 1 = Israel, 1 = Switzerland, 1 = Austria
**Locus of care**	23 = primary care, 5 = secondary care, 26 = about care or treatment in general, without specifically looking at service provision in secondary or primary care services, 2 = n/a - setting unrelated to mental health

The quality of included studies was variable, please see Additional Files 2 and 3 for assessment of relevance and rigour for the following results.

### Initial Programme theory

Following the analysis steps as listed above, including scoping searches and stakeholder meetings, initial programme theories focused heavily on GPs’ lack of knowledge and training [28–31], however most SU will know that their GP has limited training in antipsychotic management and consider that it is more important to be heard and referred at the right time [32]. Difficulties in adhering to standards were also noted in physical health [1, 5, 33, 34], suggesting that a lack of involvement in antipsychotic management may not simply be related to a lack of mental health knowledge and training alone. A lack of mental health guidance was also considered to be a potentially important factor, however, even where there was guidance available, it was not well adhered to, as seen in rates of polypharmacy for example [7, 35]. Therefore, although GP’s lack of knowledge and training are important factors, for the purposes of this review we focused on other issues.

Other factors, like the low frequency of SMI diagnoses and complex medication regimes in this population were excluded from the initial programme theory, as the former is unamenable to change and the latter is not specific to SMI diagnoses as GPs tackle complex medication management in other areas, e.g. pain management. Similarly, institutional barriers were considered to potentially play a role. Stakeholder discussions identified that GPs cannot easily identify which of their SU are primary care only, and which are also under secondary care. Although this is likely to influence the initiation of conversations about medication, it has excluded as it represents an administrative barrier that cannot be changed readily. Following the scoping searches, practice nurses were also excluded from the review, as they did not seem involved centrally [3], although there should be scope for them to be involved as recommended in the literature [36] and by the LEAP members.

Following the above listed, five step process [17, 20] five CMOCs were developed, describing potential barriers to antipsychotic medication reviews in primary care: Table 4 - CMOC Title and Sources

**Table 4:** CMOC Title and Sources

CMOC Title	Source Data used
1. Low expectations regarding recovery from mental illness	[1, 5, 30–32, 34, 37–60]
2. Perceived lack of SUs’ capabilities to participate in medication reviews	[1, 7, 23, 32, 37, 39, 44–46, 52, 54, 55, 58, 60–69]
3. Lack of information sharing between GPs and SU	[1, 5, 29, 33, 37, 43–45, 47, 54, 60, 63, 65, 66, 69–74]
4. Perceived risk of SUs	[23, 30, 39, 41–43, 47, 53, 54, 58, 59, 75–79]
5. Mutual uncertainty regarding medication and illness trajectory	[1, 4, 5, 43, 48, 49, 52, 55, 60, 66, 76, 80]

They illustrate potential explanations for a lack of conversation about, or appropriate review of antipsychotic medication in SUs diagnosed with schizophrenia or psychosis. Table 3 provides an overview of data included. They are not mutually exclusive: more than one, or none may characterise any particular situation, and each may occur to a lesser or greater extent [81]. These findings are summarised in Table 5 alongside illustrative excerpts of the data that was used to develop the CMOCs.

**Table 5:** Barriers and facilitators to antipsychotic medication reviews

Barriers	Group	CMOC	Key quote	Facilitator
**1. Low expectations**	GP	Where GPs have low expectations regarding recovery for SU diagnosed with SMI(C), and rely on antipsychotics as a main treatment (C), then they may be left feeling hopeless (M), leading to little or no ongoing antipsychotic medication reviews (O).	“the most significant obstacles to the effective management of the chronically mentally ill are the prevailing negative attitudes and believes about them” [56] - *author*	Realistic expectations of what the medication can achieve [43, 62, 66], attempt to improving QoL [52, 63]
	Service users (SU)	Where GPs communicate hopelessness to SU (C), they may in turn feel hopeless (M), and therefore unlikely to commence a conversation about medication(O).	“When I approached my GP, he [..] said, ‘Well, you’ll be on these tablets for the rest of your life,[…] being told I’d never be able to work again, I’d never have an education, never have relationships, never have anything in my life. So, for a period of time I thought well, there’s no hope” [32] – *SU focus group*	Recovery orientated treatment [78]
**2. Perceived lack of capabilities**	GP	Where GPs perceive SUs to lack capabilities and/ or “insight” (C), despite years of stability (C), GPs may act in a paternalistic/authoritarian way (M) and dismiss medication queries (O) and a conversation regarding medication(O). Additional Context: 1) Where antipsychotic side effects are apparent in SU (apathy, cognitive impairment) 2) Where GPs feel pressure to prescribe 3) Diagnostic overshadowing (see Glossary)	GPs scepticism towards reliability and insight of people with psychosis may discourage clients themselves from help-seeking, with further negative effects on their health” [59] – *author* “I’ve had difficulty in getting full regular medical check-ups as every symptom is considered a sign for stress” [47] *-SU interviews*	See SU as capable; enable SU to discuss medication/ side effects; notion that medication queries are justified [43, 44, 82, 83] Commitment to Shared Decision Making (O)
	SU	In turn, experiencing a dismissal of their queries (C), particularly if SUs have a history of being coerced to take medication or being committed to treatment against their will (sectioning) (C), this will lead to decreased trust (M) in GPs, leading SU to not discuss medication with their GP (O) and covert medication changes (O).	I think it’s just a general disregard for they have for anything that people say, because they’re mentally ill therefore you know, anything they say is questionable [..] and they say, well, I have a problem with chlorpromazine or something, they might override that, rather than listen to what the consumer is saying” [84] *– SU interview*	Feel listened to, taken seriously, time to talk [44, 82]
**3. Lack of information sharing**	SU	Information about medication: Due to a lack of information (C), SU may be unaware (M) of the risk associated with antipsychotics and the need for check-ups, leading to no conversation (O) and lack of attendance at reviews (O).	55% [of patients] said that they were unaware of the potential metabolic side-effects of atypical antipsychotic medications [..]61% said that they had had no monitoring blood tests in the past year. 69% did not know that certain monitoring blood tests were recommended [33]. – *SU response to survey*	Provide more information [33, 43, 63, 71, 85]. Research required to established what constitutes sufficient information.
	GP	Information about side effects: Where GPs are aware of side effects (C),they may fear (M) that SU will discontinue their medication (O) and feel it is in the SUs interest (M) to not share more information regarding side effects (O).	“At one time … it was … if you tell patients about side effects, they won’t take the medication.” [74] – *pharmacist interview*	Increased information sharing can lead to higher adherence and facilitates trust [60, 84]
	SU	Due to lack of discussion about side effects (C), SU may in turn feel shocked (M) and loss of trust (M), where they experience side effects (C) which may lead them to alter or discontinue medication without further consultation (O). Distrust (M) is potentially amplified when SU access information elsewhere (C), like the internet, and realise that those are potentially common side effects.	“Lack of communication about antipsychotics was the contributing factor to my stopping attempt. I recall vividly when I was sitting on the couch, watching TV, and I looked down and I noticed my chest was wet, upon further inspection I realized that I was lactating. I was shocked, scared, and terrified. It was at that moment that I decided to quit.” [63]– *SU interview*	Access to sufficient information could help to increase SU confidence to commence conversations about medication [61]
**4.Perceived risk**	GP	Despite evidence to the contrary, GPs may consider SUs to be a risk to others (C), which can lead to fear in GPs (M), which may then lead to avoidance of medication reviews (O), or GPs taking a passive role (O).	A survey of GP attitudes to people diagnosed with schizophrenia found that they endorsed either “partially true” or “completely true” for: “people are frightened by them (93.9%) and ‘they would become dangerous if they stopped their medication’ (73.9%) [59].– *GP responses to survey* A survey of provider ratings of metabolic care barriers found that the most endorsed item in the category “primary care provider barriers” is “providers are scared of people with SMI” [76]*– clinician responses to survey*	Research needed to explore how to increase GPs feeling safe in appointments.
	SU	Where SUs have current/previous experience of being perceived as frightening (C), a good GP-SU relationship or open conversation is unlikely to occur (O). We were unable to elicit a mechanism here. Mechanisms were not identified in the literature, it is possible that a loss of trust or feeling disillusioned could play a role, however further research is required.	SU “felt their GP was scared of them, ending a consultation quickly and suggesting they find a different GP” [47] – *SU interview*	Feel comfortable at their GP practice, reassurance regarding risk of being sectioned.
**5. Uncertainty regarding medication and illness trajectory**	GP	Where there is a lack of guidance and (perceived) secondary care support (C), GPs may worry (M) about relapses and lack confidence (M) in changing medication and then they may be reluctant to change medication (O) even where SU are stable in mental health (C).	Many GPs are reluctant to reduce these without supervision, especially when the patient appears well. […] There is no clear agreement on the optimum frequency for reviewing maintenance treatment, nor is there consensus on what symptom-free period warrants consideration of discontinuation [1]. *- author*	Guidance on how to review and reduce (if indicated), secondary care support [1, 43]
	SU	SU may feel equally concerned (M) to start a conversation about medication (O), due to fears of relapse (M), especially for those who have a history of sectioning (C). SU may not even be aware that medication changes are possible (C)	“This dynamic [power imbalance] resulted in some participants feeling coerced into taking medication and out of control. [..]When the option to discontinue neuroleptic medication was not explicit, participants were left with uncertainty regarding the level of support they could expect from clinicians. […] All participants acknowledged the risks of withdrawing neuroleptic medication [43]. *- SU interviews*	Continuity of care; building of trusting relationship to enable discussion of medication changes and to identify and manage potential relapse [54, 60, 82]

## Discussion

### Summary

This review set out to determine which factors influence antipsychotic medication reviews in primary care. Using realist review methodology An extensive search of the literature identified data that was used to develop several CMOCs. Taken together, the CMOCs indicate the ways in which prevalent stereotypes can impede antipsychotic medication reviews between GPs and SUs. These include:

1. low expectations of people with SMI and their recovery resulting in a lack of conversations started due to hopelessness,
2. the perception that SU lack the capabilities and “insight” required to manage their illness, leading SU to feel dismissed and not taken seriously in appointments.
3. a lack of information from both GPs and SU. GPs may not share sufficient information regarding medication risks and side effects due to fears of SU stopping medication. Equally, SU may not share all information regarding their current dose and symptoms due to fears of coercion and sectioning.
4. the perception that SU pose a risk, preventing a trusting GP – SU relationship to from forming and
5. mutual understandable concerns regarding antipsychotic medication changes, due to the potential for relapse and uncertainties regarding effects of dose reduction, resulting in avoidance of reviewing antipsychotic medication.

#### Attribution theory as substantive theory

The evidence reviewed suggested several factors that are relevant to whether appropriate medication reviews are conducted with individuals with schizophrenia or psychosis. Firstly, it identified a lack of communication between SU and GPs in relation to antipsychotic medication. Attribution theory, as substantive theory, offers a useful lens to better understand and explain the effect of these stereotypes, and potentially suggest how their effects may be reduced [23]. Attribution theory suggests that stereotypes held by clinicians and SU can change their behaviour towards each other [23, 86]. Corrigan proposes that “signals” like the label of “severe mental illness” and perceived skill deficits of mental illness can lead to a range of stereotypes, like authoritarianism and paternalism, which lead to discriminatory behaviour with regards to housing, employment and treatment. GP expectations of lack of capacity or “insight” for example can lead to paternalistic attitudes, which prevent properly informed discussions about treatment, and do not facilitate participation of the individual in the decision-making process. The review findings evidence this, as well as the need for aspects of the therapeutic relationship, like hope and trust, to counter some of those mechanisms.

#### Recommendations

**Fig. 3:**
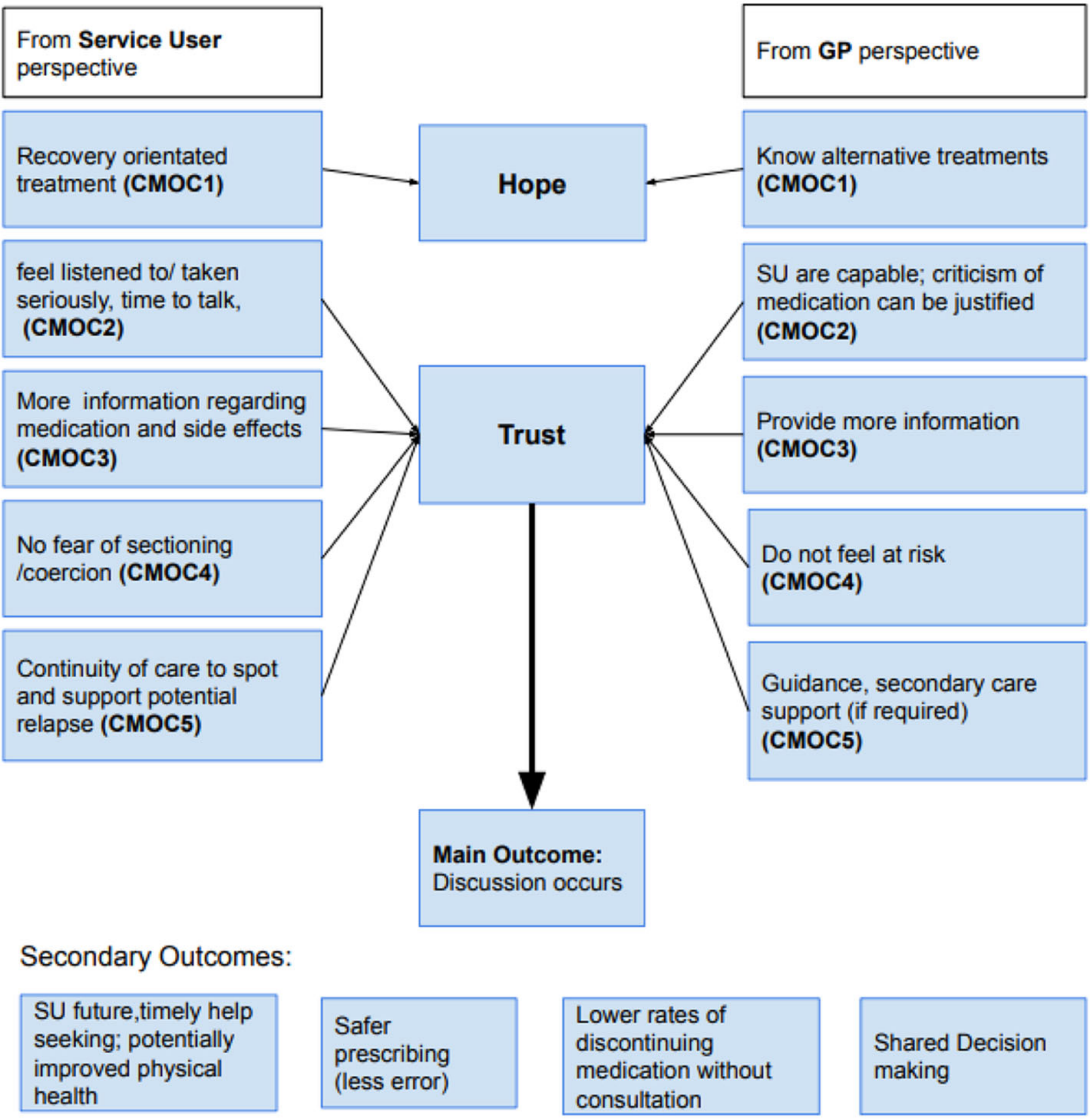
Recommendations

Using the findings above, the following recommendations can be made (Fig. 3). In order to counteract some of the mechanisms listed above, mainly hopelessness and mistrust, the following contexts have been identified as potentially counteracting the mechanism.

Increased trust has been associated with a better therapeutic alliance [43, 54, 60, 61, 82]. Given that there are multiple types of antipsychotics and dosing options, varying responses to antipsychotic medication, and no guidelines on how to review and reduce medication [16], GPs and SU encounter many uncertainties. Managing these uncertainties together requires a trusting relationship between GP and SU [60, 61]. Any history of coercion or sectioning under the Mental Health Act can make developing and maintaining trust more difficult, but a trusting relationship is key to shared decision making [29, 82]. Given the power imbalance between SU and GPs, and often held view that “doctor knows best” [82] the onus might be on the GP to start the conversation.

### Strength and limitations

This review has benefited from the input of a diverse stakeholder group, including GPs, psychiatrists, and a Lived Experience Advisory panel (LEAP). This input helped ensure that the views of these groups informed the focus of the review, and the development and refinement of the programme theory. The data included in this review was found in documents identified by a comprehensive literature search strategy, including sensitive searches in a wide range of databases and the inclusion of additional material via citation chaining. The review has been conducted and reported following the RAMESES standards [18, 19]. Conducting the review based on these guidelines included focusing the topic of the review, meaning that not every facilitator and barrier was covered in this review. Future research should address this.

The review’s findings are limited by the availability of data used to develop the CMOCs presented above. For example, no CMOCs were identified in relation to balancing risks of reducing versus continuing medication, or in relation to best methods for tapering medication when this is indicated, highlighting the need for further research in this area. The review identified little data on the involvement of other health professions, including pharmacists, in antipsychotic medication reviews. Future research should explore this role. Due to paucity of research, including pharmacists and nurses in stakeholder groups could help shed light on this.

Many of the included studies focused on specific contexts and outcomes, providing little data relating to mechanisms, or on why the outcomes they included were found. Although several included studies addressed the care of SU with a diagnosis of schizophrenia or psychosis in primary care specifically, none have researched a primary care only population. As a result, the findings are applied with caution to this population.

This review should be viewed as an initial model, which has identified several CMOCs which require further testing and refinement, eg.it is possible that there are additional C, M or Os not identified in this review.

The review also has a UK focus, and some findings may not apply to countries where GPs do not act as “gate-keepers” to secondary or specialist care.

### Comparison with existing literature

The review did not identify any existing literature, practice guidelines or interventions assessing the treatment and care of SU who are under primary care only. Previous research found that when comparing patient records, primary care only SU are older, have fewer GP appointments and are on more medication overall [2, 3]. The content of antipsychotic medication reviews, as well as their feasibility in primary care, have not been investigated. A focus group study of SU diagnosed with SMI was conducted in primary care [32], however SU were not explicitly primary care only, therefore it is difficult to estimate their treatment experiences and expectations of their interaction with GPs, since they may differ if they are no longer under secondary care. A recent systematic review also identified expectations of low capabilities, lack of trust and paternalism (including the decision to limit the amount of information regarding adverse effects shared and “doctor knows best” mentality [87];) as barriers to patient involved prescribing.

Previous literature cites negative symptoms like apathy and paranoia, as well as cognitive difficulties associated with a diagnosis of SMI, as a reason for lack of engagement with health services in this population [5, 83]. The above listed CMOCs offer additional explanations, alongside potential solutions to improve engagement in the future. A recent study on lifestyle interventions to reduce cardiovascular risk also found that primary care health professionals described people diagnosed with schizophrenia/psychosis as “threatening or scary or difficult” (p7), [88]. This prevented staff from offering interventions. These results align with the findings of this review and illustrate the impact stigma still has on service provision. Clearer guidance is needed to address issues around (perceived) risk management in this population.

### Implications for research and/or practice

More research is urgently needed to address this gap in knowledge regarding the needs of people who are currently only seen in primary care. Future research should address how GPs can be better supported to look after a population estimated to include approximately 30% of all SU with a diagnosis of schizophrenia or psychosis [2, 3]. This research should include studies linking patient-level data from primary care with secondary care patient records, to establish exact numbers of primary care only SUs, and compare the demographics and potentially unique needs of this population. Research should also explore SU and GP views on receiving or providing antipsychotic medication reviews solely in primary care. Several recommendations for practice can be made on the basis of this review’s findings. Increasing GP knowledge regarding antipsychotic treatment could help GPs to develop their confidence to balance risks and benefits and make changes to medication, like reducing doses to improve side effect burden. Greater familiarity with the recovery agenda may help GPs to appreciate the possibilities of living a fulfilling life with and without medication, to counter some of the hopelessness identified in CMOC 1.

To combat some communication difficulties (as seen in CMOC 2), GPs need to enable SU to express their views [82, 83] and take SU concerns seriously [44]. This may also include structured assessments, as SU may not volunteer problems with their medication [2]. SU complaints and queries regarding antipsychotic medication should be assumed to be justified and need proper consideration. Such ways of working are established best practice in consultation in primary care [89] but may be less common when working with individuals with psychosis. Conversations about medication should include sufficient information about antipsychotic medication (CMOC 3), and side effects as well as benefits. Increasing SU awareness of potentially severe side effects has been associated with increased trust between SU and GP [60, 82] and allows SU to prepare for side effects and return to the GP for help if they persist or cause problems. Research is needed to determine what constitutes “sufficient” information, as most data identified for this review only refers to a “lack of information” without specifying what additional information would be required.

Engagement with physical health monitoring may also increase, if SU are aware of the specific reasons for this (CMOC 3), which may tackle some health disparities between this population and the general population [70]. This may also help to avoid SU discontinuing medication without consultation. Some evidence suggests that pharmacists can help to increase knowledge [74, 84]. This could ease the pressure of time limited appointments. Access to sufficient information could help to increase SU confidence to commence conversations about medication [61], improve adherence [66, 72], patient safety [76] and facilitate Shared Decision Making (SDM [62];). A more nuanced knowledge of risk would be beneficial (CMOC 4). Whereas there are certain risks associated with a SMI diagnosis, like higher rates of substances abuse, these are not as great as perceived by the general population [58], and SU have been found to be 14 times more likely to be victims of violent crime than being the perpetrator [90]. A safe environment needs to be developed for GPs and SU alike.

Concerns about relapse are understandable (CMOC 5) but should not necessarily exclude attempts to reduce the dose of antipsychotic medication slowly and carefully to facilitate patient choice and minimise side effects and health complications. GPs may require support from secondary care for such an approach, however, and smoother liaison between primary and secondary care might be needed to facilitate this. Continuity of care has been highlighted as a crucial factor for this population [54, 82] as it can help GPs to potentially spot signs of relapse early and offer appropriate support, and is likely to be an important factor in facilitating a safe process of medication change. Continuity may also increase SU trust and encourage the start of conversations about medication. Trust could also facilitate safer prescribing [60], as SU may tailor their dose of medication, without necessarily consulting their doctor [84] and may be reluctant to disclose this due to fears of being sectioned/coerced.

Better guidance on safe reduction and discontinuation of medication [16], with a specific focus on whether this is achievable in primary care is needed, as well as better links between primary and secondary care services, as GPs do not seem to feel supported [5, 31, 91, 92]. Knowing that support is available may increase GP confidence. Alternatively, SU could be referred back to secondary psychiatric services for periodic reviews of and recommendations about their medication regimes.

This review has developed a testable programme theory highlighting the role of hope and trust in improving antipsychotic medication reviews for people diagnosed with schizophrenia and/or psychosis. Piloting and evaluating these recommendations further could be a start to strengthening trust and commencing conversations, to enable appropriate and safe prescribing, whilst also maximising quality of life.

## Supplementary Information

**Additional file 1.** Search strategy

**Additional file 2.** Data extraction tool template (adjusted from Seth Graham et al. & Jagosh et al).

**Additional file 3.** Individual CMOC quality appraisal.

**Additional file 4.** List of included papers.

## Data Availability

The datasets used and/or analysed during the current study are available from the corresponding author on reasonable request.

## References

[1] Burns T, Kendrick T (1997). The primary care of patients with schizophrenia: a search for good practice. Br J Gen Pract.

[2] Kendrick B, Freeling S. Provision of care to general practice patients with disabling long-term mental illness: a survey in 16 practices. Br J Gen Pract. 1994;44(384):301–5. PMC1238926 8068376

[3] Reilly S, Planner C, Hann M, Reeves D, Nazareth I, Lester H. The role of primary care in service provision for people with severe mental illness in the United Kingdom. PLoS One. 2012;7(5):e36468. 10.1371/journal.pone.0036468 PMC3352919 22615769

[4] Taylor D, Barnes T, Young A. The Maudsley prescribing guidelines in psychiatry. Wiley; 2018.

[5] Jones R, Major B, Fear C (2015). Schizophrenia in a primary care setting. Curr Psychiatry Rep.

[6] Raynsford J, Dada C, Stansfield D, Cullen T (2020). Impact of a specialist mental health pharmacy team on medicines optimisation in primary care for patients on a severe mental illness register: a pilot study. Eur J Hosp Pharm.

[7] Mortimer AM (2004). Atypical antipsychotics as first-line treatments for schizophrenia. Dis Manag Heal Outcomes.

[8] Miller DD. Atypical antipsychotics: sleep, sedation, and efficacy. Prim Care Companion J Clin Psychiatry [Internet] 2004;6(Suppl 2):3–7. Available from: http://www.ncbi.nlm.nih.gov/pubmed/16001094%0Ahttp://www.pubmedcentral.nih.gov/articlerender.fcgi?artid=PMC487011 PMC487011 16001094

[9] Ray WA, Chung CP, Murray KT, Hall K, Stein CM (2009). Atypical antipsychotic drugs and the risk of sudden cardiac death. N Engl J Med.

[10] Foley DL, Morley KI (2011). Systematic review of early cardiometabolic outcomes of the first treated episode of psychosis. Arch Gen Psychiatry.

[11] Moncrieff J, Leo J (2010). A systematic review of the effects of antipsychotic drugs on brain volume. Psychol Med.

[12] Fusar-Poli P, Smieskova R, Kempton MJ, Ho BC, Andreasen NC, Borgwardt S (2013). Progressive brain changes in schizophrenia related to antipsychotic treatment? A meta-analysis of longitudinal MRI studies. Neurosci Biobehav Rev.

[13] Husa AP, Rannikko I, Moilanen J, Haapea M, Murray GK, Barnett J, Jones PB, Isohanni M, Koponen H, Miettunen J, Jääskeläinen E (2014). Lifetime use of antipsychotic medication and its relation to change of verbal learning and memory in midlife schizophrenia - an observational 9-year follow-up study. Schizophr Res [Internet].

[14] Murray RM, Quattrone D, Natesan S, Van Os J, Nordentoft M, Howes O (2016). Should psychiatrists be more cautious about the long-term prophylactic use of antipsychotics. Br J Psychiatry.

[15] Morrison AP, Hutton P, Shiers D, Turkington D (2012). Antipsychotics: is it time to introduce patient choice?. Br J Psychiatry.

[16] Gupta S, Cahill JD (2016). A prescription for “deprescribing” in psychiatry. Psychiatr Serv.

[17] Pawson R. Evidence-based policy: a realist perspective: Sage; 2006. 10.4135/9781849209120.

[18] Greenhalgh T, Wong G, Westhorp G, Pawson R. Protocol - realist and meta-narrative evidence synthesis: evolving standards (RAMESES). BMC Med Res Methodol. 2011;11(1). 10.1186/1471-2288-11-115. 10.1186/1471-2288-11-115 PMC3173389 21843376

[19] Wong G, Greenhalgh T, Westhorp G, Pawson R (2014). Development of methodological guidance, publication standards and training materials for realist and meta-narrative reviews: the RAMESES (realist and meta-narrative evidence syntheses – evolving standards) project. Heal Serv Deliv Res [Internet].

[20] Pawson R, Greenhalgh T, Harvey G, Walshe K. Realist review-a new method of systematic review designed for complex policy interventions. Journal of health services research & policy. 2005 Jul;10(1_suppl):21–34. 10.1258/1355819054308530 16053581

[21] Moncrieff J, Lewis G, Freemantle N, Johnson S, Barnes TR, Morant N, Pinfold V, Hunter R, Kent LJ, Smith R, Darton K (2019). Randomised controlled trial of gradual antipsychotic reduction and discontinuation in people with schizophrenia and related disorders: the RADAR trial (research into antipsychotic discontinuation and reduction). BMJ Open.

[22] Booth A, Briscoe S, Wright JM (2020). The “realist search”: a systematic scoping review of current practice and reporting. Res Synth Methods.

[23] Corrigan PW (2000). Mental health stigma as social attribution. Clin Psychology Sci Pract.

[24] Francis-Graham S, Ekeke NA, Nelson CA, Lee TY, El Haj S, Rhodes T, Vindrola C, Colbourn T, Rosenberg W (2019). Understanding how, why, for whom, and under what circumstances opt-out blood-borne virus testing programmes work to increase test engagement and uptake within prison: a rapid-realist review. BMC Health Serv Res.

[25] Jagosh J, Pluye P, Macaulay AC, Salsberg J, Henderson J, Sirett E, Bush PL, Seller R, Wong G, Greenhalgh T, Cargo M (2011). Assessing the outcomes of participatory research: protocol for identifying, selecting, appraising and synthesizing the literature for realist review. Implement Sci.

[26] Hong Q, Pluye P, Fàbregues S, Bartlett G, Boardman F, Cargo M, et al. Mixed Methods Appraisal Tool (MMAT) Version 2018: User guide. McGill [Internet]. 2018;1–11. Available from: http://mixedmethodsappraisaltoolpublic.pbworks.com/w/file/fetch/127916259/MMAT_2018_criteria-manual_2018-08-01_ENG.pdf%0Ahttp://mixedmethodsappraisaltoolpublic.pbworks.com/.

[27] CASP (2018). Critical appraisal skills Programme. CASP Systematic Review Critical Appraisal Checklist.

[28] Baker E, Gwernan-Jones R, Britten N, Cox M, McCabe C, Retzer A (2019). Refining a model of collaborative care for people with a diagnosis of bipolar, schizophrenia or other psychoses in England: a qualitative formative evaluation. BMC Psychiatry.

[29] Boardman GH, McCann TV, Clark E (2008). Accessing health care professionals about antipsychotic medication related concerns. Issues Ment Health Nurs.

[30] Rasmussen J. Improving practice. Drugs Context [Internet] 2006;2(13):589–599. Available from: http://www.embase.com/search/results?subaction=viewrecord&from=export&id=L44842250%5Cnhttp://sfx.library.uu.nl/utrecht?sid=EMBASE&issn=17451981&id=doi:&atitle=Improving+practice&stitle=Drugs+Context&title=Drugs+in+Context&volume=2&issue=13&spage=589&epage

[31] Toews J, Lockyer J, Addington D, McDougall G, Ward R, Simpson E (1996). Improving the management of patients with schizophrenia in primary care: assessing learning needs as a first step. Can J Psychiatr.

[32] Lester HE, Tritter JQ, Sorohan H (2005). Patients’ and health professionals’ views on primary care for people with serious mental illness: focus group study. Br Med J.

[33] Feeney L, Mooney M (2006). Atypical antipsychotic monitoring: a survey of patient knowledge and experience. Ir J Psychol Med.

[34] Lambert TJR, Newcomer JW (2009). Are the cardiometabolic complications of schizophrenia still neglected? Barriers to care. Med J Aust.

[35] Patel MX, Bishara D, Jayakumar S, Zalewska K, Shiers D, Crawford MJ, Cooper SJ (2014). Quality of prescribing for schizophrenia: evidence from a national audit in England and Wales. Eur Neuropsychopharmacol [Internet].

[36] Millar E, Garland C, Ross F, Kendrick T, Burns T (1999). Practice nurses and the care of patients receiving depot neuroleptic treatment: views on training, confidence and use of structured assessment. J Adv Nurs [Internet].

[37] Morant N, Kaminskiy E, Ramon S (2016). Shared decision making for psychiatric medication management: beyond the micro-social. Health Expect.

[38] Rogers A, Campbell S, Gask L, Sheaff R, Marshall M, Halliwell S, Pickard S (2002). Some National Service Frameworks are more equal than others: implementing clinical governance for mental health in primary care groups and trusts. J Ment Health.

[39] Royal College of Psychiatrists. Mental illness : stigmatisation and discrimination within the medical. 2001. 1–40 p.

[40] Hustig HH, Norrie PD (1998). Managing schizophrenia in the community. Med J Aust.

[41] Dixon RP, Roberts LM, Lawrie S, Jones LA, Humphreys MS (2008). Medical students’ attitudes to psychiatric illness in primary care. Med Educ.

[42] Schulze B (2007). Stigma and mental health professionals: a review of the evidence on an intricate relationship. Int Rev Psychiatry.

[43] Le Geyt G, Awenat Y, Tai S, Haddock G (2017). Personal accounts of discontinuing neuroleptic medication for psychosis. Qual Health Res.

[44] Lambert TJR, Chapman LH, Bell S, Carr N, D’Emden M, Elsom S (2004). Diabetes, psychotic disorders and antipsychotic therapy: a consensus statement. Med J Aust.

[45] Pereira S, Pinto R. A survey of the attitudes of chronic psychiatric patients living in the community toward their medication. Acta Psychiatr Scand [Internet]. 1997;95(6):464–468. Available from: http://ovidsp.ovid.com/ovidweb.cgi? T=JS&PAGE=reference&D=emed4&NEWS=N&AN=1997193220. 10.1111/j.1600-0447.1997.tb10133.x 9242840

[46] Tranulis C, Goff D, Henderson DC, Freudenreich O. Becoming adherent to antipsychotics: a qualitative study of treatment-experienced schizophrenia patients. Psychiatr Serv. 2011;62(8):888–92. 10.1176/ps.62.8.pss6208_0888. 10.1176/ps.62.8.pss6208_0888 21807827

[47] Pilgrim D, Rogers A (1993). Mental health service users’ views of medical practitioners. J Interprof Care.

[48] Kendrick T, Burns T, Freeling P (1995). Randomised controlled trial of teaching general practitioners to carry out structured assessments of their long term mentally ill patients. Bmj..

[49] Mortimer A., Shepherd C., Rymer M, Burrows A. Primary care use of antipsychotic drugs: An audit and intervention study. Ann Gen Psychiatry. 2005;4(1). 10.1186/1744-859X-4-18 PMC1318453 16316473

[50] Galon P, Heifner GC (2012). Engagement in primary care treatment by persons with severe and persistent mental illness. Arch Psychiatr Nurs.

[51] Donlon PT (1978). The schizophrenias: medical diagnosis and treatment by the family physician. J Fam Pract.

[52] Morrison P, Meehan T, Stomski NJ (2015). Living with antipsychotic medication side-effects: the experience of Australian mental health consumers. Int J Ment Health Nurs.

[53] Lawrie M, McNeill D, Chrystie R (1998). General practitioners’ attitudes to psychiatric and medical illness. Psychol Med.

[54] Lester H, Tritter JQ, England E (2003). Satisfaction with primary care: the perspectives of people with schizophrenia. Fam Pract.

[55] Johnson DAW, Rasmussen JGC (1997). Professional attitudes in the UK towards neuroleptic maintenance therapy in schizophrenia. Psychiatr Bull.

[56] Jones LR, Knopke HJ. Educating Family Physicians To Care for the Chronically Mentally Ill. 1987;24(2):177–83. 2879879

[57] Viron M, Baggett T, Hill M, Freudenreich O (2012). Schizophrenia for primary care providers: how to contribute to the care of a vulnerable patient population. Am J Med [Internet].

[58] Katschnig H (2018). Psychiatry’s contribution to the public stereotype of schizophrenia: historical considerations. J Eval Clin Pract.

[59] Magliano L, Strino A, Punzo R, Acone R, Affuso G, Read J (2017). Effects of the diagnostic label “schizophrenia”, actively used or passively accepted, on general practitioners’ views of this disorder. Int J Soc Psychiatry.

[60] Maidment ID, Brown P, Calnan M (2011). An exploratory study of the role of trust in medication management within mental health services. Int J Clin Pharm.

[61] Delman J, Clark JA, Eisen SV, Parker VA (2015). Facilitators and barriers to the active participation of clients with serious mental illnesses in medication decision making: the perceptions of young adult clients. J Behav Health Serv Res.

[62] Roe D, Goldblatt H, Baloush-Klienman V, Swarbrick M, Davidson L (2009). Why and how people decide to stop taking prescribed psychiatric medication: exploring the subjective process of choice. Psychiatr Rehabil J..

[63] Salomon C, Hamilton B (2013). “All roads lead to medication?” qualitative responses from an Australian first-person survey of antipsychotic discontinuation. Psychiatr Rehabil J.

[64] Rogers A, Day JC, Williams B, Randall F, Wood P, Healy D (1998). The meaning and management of neuroleptic medication: A study of patients with a diagnosis of schizophrenia. Soc Sci Med [Internet].

[65] Seale C, Chaplin R, Lelliott P, Quirk A (2007). Antipsychotic medication, sedation and mental clouding: an observational study of psychiatric consultations. Soc Sci Med.

[66] Britten N, Riley R, Morgan M (2010). Resisting psychotropic medicines: a synthesis of qualitative studies of medicine-taking. Adv Psychiatr Treat.

[67] Leucht S, Arbter D, Engel RR, Kissling W, Davis JM (2009). How effective are second-generation antipsychotic drugs? A meta-analysis of placebo-controlled trials. Mol Psychiatry.

[68] Usher K. Taking neuroleptic medications as the treatment for schizophrenia: a phenomenological study. Aust N Z J Ment Health Nurs [Internet] 2001;10(3):145–155. Available from: 10.1046/j.1440-0979.2001.00205.x/abstract%5Cnhttp://onlinelibrary.wiley.com/doi/10.1046/j.1440-0979.2001.00205.x/full%5Cnhttp://onlinelibrary.wiley.com/doi/10.1046/j.1440-0979.2001.00205.x/pdf 10.1046/j.1440-0979.2001.00205.x 11493286

[69] Carrick R, Mitchell A, Powell RA, Lloyd K (2004). The quest for well-being: a qualitative study of the experience of taking antipsychotic medication. Psychol Psychother Theory Res Pract.

[70] Crawford MJ, Jayakumar S, Lemmey SJ, Zalewska K, Patel MX, Cooper SJ, Shiers D (2014). Assessment and treatment of physical health problems among people with schizophrenia: national cross-sectional study. Br J Psychiatry.

[71] Aref-Adib G, O’Hanlon P, Fullarton K, Morant N, Sommerlad A, Johnson S, Osborn D (2016). A qualitative study of online mental health information seeking behaviour by those with psychosis. BMC Psychiatry [Internet].

[72] Mitchell AJ, Selmes T (2007). Why don’t patients take their medicine? Reasons and solutions in psychiatry. Adv Psychiatr Treat.

[73] Schachter D, Kleinman I, Williams JI (1999). Informed consent for antipsychotic medication: Do family physicians document obtaining it?. Can Fam Physician.

[74] Younas M, Bradley E, Holmes N, Sud D, Maidment ID (2016). Mental health pharmacists views on shared decision-making for antipsychotics in serious mental illness. Int J Clin Pharm.

[75] Oud MJ, Schuling J, Slooff CJ, Groenier KH, Dekker JH, Meyboom-De JB (2009). Care for patients with severe mental illness: the general practitioner’s role perspective. BMC Fam Pract.

[76] McDonell MG, Kaufman EA, Srebnik DS, Ciechanowski PS, Ries RK (2011). Barriers to metabolic Care for Adults with serious mental illness: provider perspectives. Int J Psychiatry Med.

[77] Corrigan PW, Kosyluk KA (2013). Erasing the stigma: where science meets advocacy. Basic Appl Soc Psych.

[78] The Schizophrenia Commission. The Abandoned Illness: A Report by the Schizophrenia Commission [Internet]. 2012. London: Rethink Mental Illness. Available from: http://www.healthpromoting.com/sites/default/files/fastingsp2011.pdf

[79] Dillner L (1995). Mental health law obsolete, says inquiry. BMJ [Internet].

[80] Carr VJ, Lewin TJ, Barnard RE, Walton JM, Allen JL, Constable PM, Chapman JL (2004). Attitudes and roles of general practitioners in the treatment of schizophrenia compared with community mental health staff and patients. Soc Psychiatry Psychiatr Epidemiol.

[81] Dalkin SM, Greenhalgh J, Jones D, Cunningham B, Lhussier M (2015). What’s in a mechanism? Development of a key concept in realist evaluation. Implement Sci.

[82] Joseph-Williams N, Elwyn G, Edwards A (2014). Knowledge is not power for patients: a systematic review and thematic synthesis of patient-reported barriers and facilitators to shared decision making. Patient Educ Couns [Internet].

[83] Annamalai A, Tek C (2015). An overview of diabetes Management in Schizophrenia Patients: office based strategies for primary care practitioners and endocrinologists. Int J Endocrinol.

[84] Happell B, Manias E, Rope C. Wanting to be heard: mental health consumers' experience of information about medication. Int J Ment Health Nurs. 2004;13(4):242–8. 10.1111/j.1440-0979.2004.00340.x 15660592

[85] Happell B, Platania-Phung C, Scott D (2014). Proposed nurse-led initiatives in improving physical health of people with serious mental illness: a survey of nurses in mental health. J Clin Nurs.

[86] Corrigan P (2004). How stigma interferes with mental health care. Am Psychol.

[87] Pedley R, McWilliams C, Lovell K, Brooks H, Rushton K, Drake RJ, Rumbold B, Bell V, Bee P (2018). Qualitative systematic review of barriers and facilitators to patient-involved antipsychotic prescribing. BJPsych Open.

[88] Burton A, Osborn D, Atkins L, Michie S, Gray B, Stevenson F (2015). Lowering cardiovascular disease risk for people with severe mental illnesses in primary care: a focus group study. PLoS One.

[89] Pendleton D. The consultation: an approach to learning and teachingle. Oxford University Press; 1984.

[90] Brekke JS, Prindle C (2001). Sung woo Bae, long JD. Risks for individuals with schizophrenia who are living in the community. Psychiatr Serv.

[91] Carr VJ (1997). The role of the general practitioner in the treatment of schizophrenia: specific issues. Med J Aust.

[92] Bindman J, S. J, S. W, G. S, P. B, E. K. Integration between primary and secondary services in the care of the severely mentally ill: Patients’ and general practitioners’ views. Br J Psychiatry [Internet]. 1997;171(AUG.):169–74. Available from: https://www.ncbi.nlm.nih.gov/pubmed/9337955. 10.1192/bjp.171.2.169 9337955

