# Supplementary material for: The role of trust and hope in antipsychotic medication reviews between GPs and service users a realist review
Source: BMC Psychiatry. 2021 Aug 4;21:390. doi: 10.1186/s12888-021-03355-3 (PMC8340528; doi:10.1186/s12888-021-03355-3)
Supplement: Supplementary file 1 — Additional file 1. Search strategy [file 12888_2021_3355_MOESM1_ESM.docx]

### 1. Additional File: Search strategy

**1.2 Main Search Strategy**

**: ~"**(((antipsychotic*).ti,ab OR (exp "TRANQUILIZING AGENTS"/ OR exp "ANTIPSYCHOTIC AGENTS"/) OR (anti-psychotic*).ti,ab OR (neuroleptic*).ti,ab OR ("major tranquiliser*").ti,ab OR ("major tranquilizer*").ti,ab OR (atypical*).ti,ab) AND (("general practice*").ti,ab OR ("general practitioner*").ti,ab OR exp PHYSICIANS/ OR exp "GENERAL PRACTITIONERS"/ OR exp "GENERAL PRACTICE"/ OR exp "GENERAL PRACTICE"/ OR exp "GENERAL PRACTITIONERS"/ OR (GP*).ti,ab OR ("family practice*").ti,ab OR exp "GENERAL PRACTICE"/ OR exp "FAMILY PRACTICE"/ OR (physician*).ti,ab OR *PHYSICIANS/ OR *"GENERAL PRACTITIONERS"/ OR *PHYSIATRISTS/ OR *"PHYSICIANS, FAMILY"/ OR *"PHYSICIANS, PRIMARY CARE"/ OR ("primary care").ti,ab OR exp "PRIMARY HEALTH CARE"/ OR ("primary health care").ti,ab OR (pharmacy).ti,ab OR *PHARMACY/ OR *"PHARMACY RESEARCH"/ OR exp *"COMMUNITY PHARMACY SERVICES"/ OR (pharmacist*).ti,ab OR exp PHARMACISTS/ OR ("nurse prescriber").ti,ab)) [DT 1954-2018] [Human age groups Young adult OR Adult OR Middle Aged OR Aged OR Aged,80 and over] [Languages English] [Humans]**"**

**1.2 Iterative Search Strategy – conducted in google scholar**

(GP OR General practitioner OR Primary care) AND (stigma OR stereotype) AND (severe mental illness OR SMI OR schizophrenia OR psychosis)
