# Supplementary material for: The role of trust and hope in antipsychotic medication reviews between GPs and service users a realist review
Source: BMC Psychiatry. 2021 Aug 4;21:390. doi: 10.1186/s12888-021-03355-3 (PMC8340528; doi:10.1186/s12888-021-03355-3)
Supplement: Supplementary file 2 — Additional file 2. Data extraction tool template (adjusted from Seth Graham et al. & Jagosh et al). [file 12888_2021_3355_MOESM2_ESM.docx]

### 2. Additional File : Data extraction tool template (adjusted from Seth Graham et al & Jagosh et al).

###
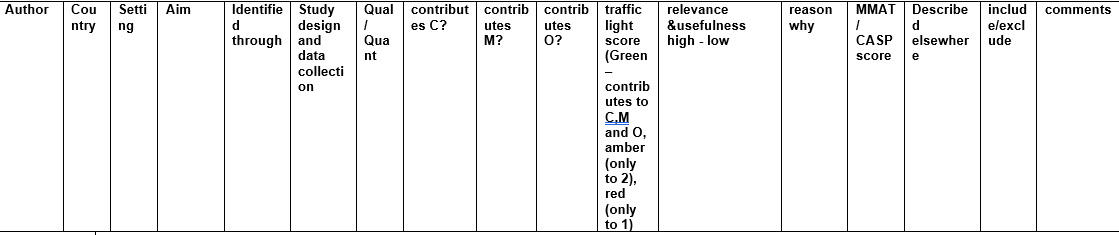


### Overall Quality Appraisal

**Overall quality appraisal** – sorted by quality appraisal tool.

Table 2 Papers assessed using CASP

| First author | country | setting | Aim | Study Design and data collection | Traffic light score | Relevance & Usefulness | S1 | S2 | 1 | 2 | 3 | 4 | 5 | 6 | 7 | 8 |
| --- | --- | --- | --- | --- | --- | --- | --- | --- | --- | --- | --- | --- | --- | --- | --- | --- |
| Britten, 2010 | UK | SC | Describe lay perspectives on prescribed psychotropic medicines. | Systematic review of qualitative studies | Green  (C,M,O) | High | y | y | y | n | y | y | y | y | n | n/a |

Table 3 Papers assessed using MMAT

| First author | country | setting | Aim | Study Design and data collection | Traffic light score | Relevance & Usefulness | Qual Appraisal | S1 | S2 | 1 | 2 | 3 | 4 | 5 |
| --- | --- | --- | --- | --- | --- | --- | --- | --- | --- | --- | --- | --- | --- | --- |
| Adams,  2007 | USA | SC | Perceived roles and preferences were explored for shared decision making among persons with severe mental illnesses. | Questionnaire | Amber (C,O) | Low | MMAT | y | y | y | y | y | n | y |
| Aref-Adib, 2016 | UK | SC | To explores the nature, extent and consequences of online mental health information seeking behaviour by people with psychosis and to investigate the acceptability of a mobile mental health application (app). | Qualitative interviews | Green (C,M,O) | Moderate | MMAT | y | y | y | y | y | y | y |
| Boardman, 2008 | Australia | SC + GP | To describe SUs’ access to and satisfaction with health care professionals, including nurses, as related to users’ antipsychotic medication concerns. | Questionnaire | Green  (C,M,O) | High | MMAT | y | y | y | m | y | m | y |
| Carr, 2004 | Australia | PC | To examines the attitudes and roles of Australian GPs in the treatment of schizophrenia and their relationships with specialist services. | Questionnaires (completed by GPs, mental health staff and service users) | Green  (C,M,O) | Moderate | MMAT | y | y | y | y | y | m | ? |
| Carrick, 2004 | UK | SC | To outline the experience of taking antipsychotic medication | Qualitative interviews + focus group | Amber (C,M) | Moderate | MMAT | y | y | y | y | y | m | y |
| Crawford, 2014 | UK | SC + GP | To examine the quality of assessment and treatment of physical health problems in people with schizophrenia. | Audit of routine data + questionnaire | Amber  (C,O) | Low | MMAT | Y | y | y | y | y | n | y |
| Delman, 2015 | USA | SC | To explore factors influencing active participation of young SU in psychotropic medication decision making | Qualitative interviews | Green (C,M,O) | High | MMAT | y | y | y | y | y | y | y |
| Dixon, 2008 | UK | PC | We describe a study of the attitudes and predicted behaviours of medical students towards patients with mental illness in primary care. To investigate the effects that level of undergraduate medical training and personal characteristics might have on responses. | Vignettes (either schizophrenia, depression, diabetes or no illness) and questionnaire | Amber (C,O) | Moderate | MMAT | y | y | y | y | y | y | y |
| Feeney, 2006 | Ireland | SC | To examine the knowledge and experiences of side-effects and their monitoring in patients prescribed atypical antipsychotic medications. | Questionnaire | Green (C,M,O) | Moderate | MMAT | Y | Y | Y | Y | Y | Y | y |
| Galon, 2012 | USA | PC | To describe the social process of engagement in primary care treatment from the perspective of persons with SPMI. | Qualitative interviews | Green  (C,M,O) | Moderate | MMAT | y | y | y | y | y | y | y |
| Happell, 2004 | Australia | SC | To examine the experiences of consumers, specifically in relation to education and decision making with regards to medication. | Focus group | Green  (C,M,O) | High | MMAT | y | y | y | y | y | y | Y |
| Johnson, 1997 | UK | mixed | To assess length of time considered suitable for treatment of schizophrenia | Teleconference between consultant psychiatrists, GPs, pharmacists and CPNs + Questionnaire + commentary | Green  (C,M,O) | Moderate | MMAT | y | y | y | y | y | Y | n |
| Kendrick, 1995 | UK | PC | To assess the impact of teaching general practitioners to carry out structured assessments of their long term mentally ill patients. | RCT of structured assessments vs TAU | Green  (C,M,O) | Moderate | MMAT | y | y | n | y | y | n | Y |
| Lawrie, 1998 | UK | PC | To examine the attitudes of general practitioners to patients with diﬀerent psychiatric and medical illnesses. | Vignettes | Green  (C,M,O) | High | MMAT | y | y | y | y | n | y | y |
| LeGeyt, 2016 | UK | SC | To explore personal accounts of making choices about taking medication prescribed for the treatment of psychosis (neuroleptics). | Qualitative Interviews | Green  (C,M,O) | High | MMAT | y | y | y | y | y | y | Y |
| Lester, 2005 | UK | PC | To explore the experience of providing and receiving primary care from the perspectives of primary care health professionals and patients with SMI respectively | Focus group | Green  (C,M,O) | High | MMAT | y | y | y | y | y | y | Y |
| Lester, 2003 | UK | PC | This study aimed to explore the elements of satisfaction with primary care for people with schizophrenia. | Qualitative interviews | Green  (C,M,O) | High | MMAT | y | y | y | y | y | y | y |
| Magliano, 2017 | Italy | PC | To investigate GPs’ views of schizophrenia and whether they were influenced by a ‘schizophrenia’ label, passively accepted or actively used. | Vignette + Questionnaire |  | High | MMAT | y | y | y | y | y | y | y |
| Maidment, 2011 | UK |  | To develop understandings of the nature and inﬂuence of trust in the safe management of medication within mental health services | Focus groups | Green  (C,M,O) | High | MMAT | y | y | y | y | y | y | y |
| McDonnell, 2011 | USA | PC | This study assessed barriers to metabolic care for persons with serious mental illness (SMI) by surveying experienced healthcare providers. | Questionnaire | Green  (C,M,O) | Moderate | MMAT | y | y | y | y | y | y | y |
| Mortimer, 2005 | UK | PC | To audit and intervene in the suboptimal prescribing of antipsychotic drugs to primary care patients. | Audit + intervention study | Amber (C,O) | Moderate | MMAT | y | y | y | ? | y | n | Y |
| Morrison, 2015 | Australia | SC | The present study explores people’s experience of living with antipsychotic medication side-effects | Qualitative interview | Green (C,M,O) | High | MMAT | y | y | y | y | y | n | y |
| Oud, 2009 | UK | PC | Responsibility and nature of care for people with SMI was explored from a GP perspective | Questionnaire | Amber (C,O) | Moderate | MMAT | y | y | y | y | y | n | y |
| Pereira, 1997 | UK | SC | To assess the acceptability of depot among those patients receiving medication via this route and, finally, to assess the views of subjects receiving oral medication about depot. | Questionnaire | Amber  (C,O) | Moderate | MMAT | y | y | y | y | y | y | y |
| Pilgrim, 1993 | UK | PC | positive and negative views about general practitioners (GPs) and psychiatrists are examined. | Questionnaire (with open ended Q) | Green  (C,M,O) | High | MMAT | y | y | y | y | y | y | ct* |
| Roe,  2009 | Israel | SC | The purpose of the present study was to explore why and how people with a serious mental illness (SMI) choose to stop taking prescribed medication | Qualitative interviews | Green  (C,M,O) | Moderate | MMAT | y | y | y | y | y | y | y |
| Rogers,  1998 | UK | SC | To describe the meaning and management of neuroleptic medication by people who have received a diagnosis of schizophrenia. | Qualitative interviews | Green  (C,M,O) | High | MMAT | y | y | y | y | y | ? | n |
| Salomon,  2013 | Australia | SC | The purpose of the survey was to better understand the experiences of people who attempt antipsychotic discontinuation. | Questionnaire | Green  (C,M,O) | Moderate | MMAT | y | y | y | y | y | y | y |
| Schachter, 1999 | Canada | PC | To educate about informed consent | Survey | Amber  (C,M) | Moderate | MMAT | y | y | y | y | y | y | y |
| Seale,2007 | UK | SC | To explore how discussions about side effects are managed in practice | Observational study + Conversation Analysis | Green  (C,M,O) | Moderate | MMAT | y | y | y | y | y | y | y |
| Toews,  1996 | Canada | PC | To assess family physician learning needs related to the care of patients with schizophrenia. | Questionnaire | Green  (C,M,O) | Moderate | MMAT | y | y | y | ? | y | n | y |
| Tranulis,  2011 | Canada | SC | To explore views on illness and medication use and emphasized key turning points, such as periods of nonadherence and illness relapses. | Qualitative interviews | Green  (C,M,O) | Moderate | MMAT | y | y | y | y | y | y | Y |
| Usher, 2001 | Australia | SC | To explore the experience of taking neuroleptic medications from the individual’s perspective | Qualitative interviews | Green  (C,M,O) | Moderate | MMAT | y | y | y | y | y | y | y |
| Younas,  2016 | UK | PC | To explore the views and experiences of UK mental health pharmacists regarding the use of SDM in antipsychotic prescribing in people diagnosed with SMI. | Qualitative Interviews | Green  (C,M,O) | Moderate | MMAT | y | y | y | y | y | y | y |

- ct = can’t tell

Table ‑4 Non quality assessed papers

| First author | country | setting | Aim | Study Design and data collection | Traffic light score | Relevance & Usefulness |
| --- | --- | --- | --- | --- | --- | --- |
| BMJ News, 1995 | UK | SC | news report | News report | Green  (C,M,O) | Moderate |
| Burns, 1997 | UK | PC | To develop practice for establishing a register and organizing regular reviews; comprehensive assessments; information and advice for patients and carers; indications for involving specialist services; and crisis management. | Consensus group developed good practice guidelines based on current literature | Green (C,M,O) | High |
| Corrigan, 2000 | USA | G | To illustrate how attribution model advances research questions related to mental health stigma | Non-systematic literature review | Green (C,M,O) | High |
| Corrigan, 2013 | USA | G | Review of existing research regarding public stigma reduction, looking at approaches within mental health and other stigmatised communities. | Non-systematic literature review | Green (C,M,O) | High |
| Donlon,1987 | USA | PC | Overview of care of schizophrenia in primary care | Non – systematic literature review | Amber  (C,O) | Moderate |
| Hustig, 1998 | Australia | PC | Overview of care of schizophrenia in primary care | MJA Practice Essentials (non systematic literature review) | Amber (C,M) | low |
| Jones, 1987 | USA | PC | overview of care of schizophrenia in primary care | Non – systematic literature review | Amber  (C,M) | Moderate |
| Jones, 2015 | UK (but studies from all over) | PC | overview of care of schizophrenia in primary care | Non – systematic literature review | Green  (C,M,O) | High |
| Katschnig, 2018 | Austria | SC | To discuss the origins of the idea of a chronic brain disease, of the split personality concept derived from the term “schizophrenia” , and the craziness idea reflected in the “first rank symptoms”, which are all hallucinations and delusions . | Non – systematic literature review | Amber (C –“split personality”, Lack of expectations, M fear) | Moderate |
| Lambert, 2009 | USA mostly | PC | barriers of physical health testing in primary care | Non systematic literature review | Green  (C,M,O) | Moderate |
| Royal College of Psychiatrists | UK | SC | Report to combat and reduce stigmatisation of people with mental disorders. | Non – systematic literature review | Amber (C,M) | Moderate |
| Mitchel & Selmes, 2007 | UK | SC | To discuss patients’ reasons for failure to concord with medical advice, and predictors of and solutions to the problem of nonadherence. | Non – systematic literature review | Green  (C,M,O) | Moderate |
| Morant, 2016 | UK | SC | This conceptual review argues that several aspects of mental health care that diﬀer from other health-care contexts may impact on processes and possibilities for SDM. | Conceptual review | Green  (C,M,O) | High |
| Mortimer, 2004 | UK | PC | Review on antipsychotic prescribing | Non – systematic literature review | Green  (C,M,O) | Low |
| NICE, 2014 | UK | SC | Guidelines on treatment and management | Evidence based guideline | Amber (C,O) | Low |
| Rasmussen2006 | UK | PC | Overview of care of people with SMI for GPs | Non – systematic literature review | Green  (C,M,O) | High |
| Schizophrenia Commission, 2012 | UK | G | To examine the provision of  care for people living with psychotic illness. | Non-systematic literature review + survey + visits to services | Amber (C,M) | Low |
| Schulze,  2017 | Switzerland | SC | To explore ways in which mental health professionals are  ‘entangled’ in anti-stigma activities. It will outline the complex relationships between stigma and the psychiatric profession,  presenting evidence on how its members can stigmatizers, stigma recipients and powerful agents of de-stigmatization. | Non – systematic literature review | Green  (C,M,O) | Moderate |
| Viron,2012 | USA | PC | This review provides primary care providers with a general understanding of the psychiatric and medical issues speciﬁc to patients with schizophrenia and a clinically practical framework for engaging and assessing this vulnerable patient population | Non- systematic literature review | Green  (C,M,O) | Moderate |
