# Supplementary material for: The role of trust and hope in antipsychotic medication reviews between GPs and service users a realist review
Source: BMC Psychiatry. 2021 Aug 4;21:390. doi: 10.1186/s12888-021-03355-3 (PMC8340528; doi:10.1186/s12888-021-03355-3)
Supplement: Supplementary file 3 — Additional file 3. Individual CMOC quality appraisal. [file 12888_2021_3355_MOESM3_ESM.docx]

### 3. Additional File: Individual CMOC quality appraisal

Following the development of specific Context, Mechanism and Outcome Configurations (CMOCs, see Glossary), each was quality assessed. The overall quality of the data included in the development of each CMOC was considered in relation to several criteria. For each CMOC, included data was first assessed in relation to its contribution of information relating to C, M or O (as above). Scores were given accordingly: A - papers providing evidence on C, M and O relevant to this CMOC, B - papers providing evidence on only two of the three (C, M, O) criteria or C - providing information only on one of C, M or O). Secondly, relevance was further assessed on a 3 point scale of A to C: A - papers of high relevance to the CMOC, B- papers of moderate relevance to the CMOC and C- low relevance. The closer the data was to discussing C,M or O in primary care, for people diagnosed with SMI and treated with antipsychotics, the higher the relevance. Reasons for each scoring was recorded for transparency. Thirdly, the quality of the evidence was assessed, again on a A-C scale: A - evidence was derived directly through the studies’ findings B -evidence was taken from the discussion, based on the study’s findings (this allows for the inclusion of the authors suggestions on the nature of their findings) and C - taken from the introduction or from a non-systematic literature review, opinion or editorial. LG completed this assessment for each individual CMOC, any queries were discussed with CD and resolved by discussion.

| **First author, year** | **Country** | **Setting** | **Aim** | **Study design and data collection** | **Dimensions of relevance** | **Strength of relevance** | **Methodological quality** |
| --- | --- | --- | --- | --- | --- | --- | --- |
| Britten, 2010 | UK | SC | Describe lay perspectives on prescribed psychotropic medicines. | Systematic review of qualitative studies | B ( C lack of info M uncertainty) | B moderately relevant, not AP specific | A taken from findings |
| Britten, 2010 | UK | SC | Describe lay perspectives on prescribed psychotropic medicines. | Systematic review of qualitative studies | A (C- lack of info given, M - worries about medication, assess pros and cons but with incomplete info O- stopping abruptly (as they have incomplete knowledge) | A - v relevant, little on GPs action though | B - taken from intro and from findings |
| Burns, 1997 | UK | PC | To develop practice for establishing a register and organizing regular reviews; comprehensive assessments; information and advice for patients and carers; indications for involving specialist services; and crisis management. | Consensus group developed good practice guidelines based on current literature | ( C need for individual treatment, stable after a few years, lack of guidance, M - GPs feel uncomfortable O - no med change without secondary care, C uncertainty as to how long to continue meds for, need for continuous reviews of medication | B relevant, shows some of the uncertainty, thin on mechanisms | C non systematic literature review |
| Carr, 2004 | Australia | PC | To examines the attitudes and roles of Australian GPs in the treatment of schizophrenia and their relationships with specialist services. | Questionnaires (completed by GPs, mental health staff and service users) | A( M - uncomfortable, lacking confidence, O - reluctant to treat, C not my responsibility, MH medication is more specialist work) | C low relevance | B - take from mixture of intro, findings and discussion |
| Carrick, 2004 | UK | SC | To outline the experience of taking antipsychotic medication | Qualitative interviews + focus group | B (C - unpredictable illness, side effects, SU and doctors in same uncertainty, M - lucky to be well, M mistrust in medical institution) | B relevant but not GP specific | A - taken from findings |
| Donlon,1987 | USA | PC | Overview of care of schizophrenia in primary care | Non – systematic literature review | C - close monitoring, reduce dose after a while, rough guidance, need to know relapse symptoms to reinstate medication, chronic illness and limitations, need empathy | C low | C non systematic literature review |
| Happell, 2004 | Australia | SC | To examine the experiences of consumers, specifically in relation to education and decision making with regards to medication. | Focus group | C misconception about being unwell, blame SU, GP have different priorities to SU, O -SU manage own medication without consultation, M - not listened to/blamed, M fear of repercussions | B relevant but not GP specific | A taken from findings |
| Johnson, 1997 | UK | mixed | To assess length of time considered suitable for treatment of schizophrenia | Teleconference between consultant psychiatrists, GPs, pharmacists and CPNs + Questionnaire + commentary | C (unable to say who can come off meds) | B relevant, illustrates uncertainty | C - taken from discussion |
| Jones, 2015 | UK (but studies from all over) | PC | overview of care of schizophrenia in primary care | Non – systematic literature review | C (lack of confidence managing SMI) | C- low relevance, unclear what O and M is | C non systematic lit review |
| LeGeyt, 2017 | UK | SC | To explore personal accounts of making choices about taking medication prescribed for the treatment of psychosis (neuroleptics). | Qualitative Interviews | C( C - GP does not feel comfortable, no option but doing it on your own, discontinuation not an option, wishing for alternatives, lack of communication) | B - relevant, but little on O and M | A finding from study |
| Maidment, 2011 | UK | SC | To develop understandings of the nature and inﬂuence of trust in the safe management of medication within mental health services | Focus groups | A (C uncertainty, M - affects trust O doctors do not consider reduction possible, relapse is possible with reduction of dose) | B - relevant, but little on M | A finding from study |
| Morant, 2016 | UK | SC | This conceptual review argues that several aspects of mental health care that diﬀer from other health-care contexts may impact on processes and possibilities for SDM. | Conceptual review | B (C - risk adverse culture, favouring relapse avoidance over issues with long term medication, M lack of confidence in stopping or reducing) | B relevant, but little on GP | C non systematic lit review |
| Mortimer, 2004 | UK | PC | Review on antipsychotic prescribing | Non – systematic literature review | B (no gain in changing things? scared of what might happen/rocking the boat esp. in this group of people) | A – valuable M | C non specific lit review |
| Morrison, 2015 | Australia | SC | The present study explores people’s experience of living with antipsychotic medication side-effects | Qualitative interview | A (C requests to change medication as way to deal with side effects, doctors dissuade, M Su just resigned to taking meds, O - nothing changes C coercion? F- nurses could help rebalance) | B relevant, different to papers from uk | A largely taken from study findings |
| Mortimer, 2005 | UK | PC | To audit and intervene in the suboptimal prescribing of antipsychotic drugs to primary care patients. | Audit + intervention study | A( C - CPN as other agent, M -fear of relapse in staff despite evidence that medication was inappropriate O - medication wasn’t taken) | B moderate relevance | A taken from findings |
| Roe,  2009 | Israel | SC | The purpose of the present study was to explore why and how people with a serious mental illness (SMI) choose to stop taking prescribed medication | Qualitative interviews | A ( M - fear that there will be repercussions, question sanity, O - changes made alone C stigma) | C low | A taken from findings |
| Seale,2007 | UK | SC | To explore how discussions about side effects are managed in practice | Observational study + Conversation Analysis | C- power imbalance, O concern not taken seriously, M question ability to say that reduction in medication might be appropriate | B - not GP | A from findings |
| Seale,2007 | UK | SC | To explore how discussions about side effects are managed in practice | Observational study + Conversation Analysis | C -reductions are happening, but it's secondary care | C low | A - taken from findings |
| Usher | Australia | SC | To explore the experience of taking neuroleptic medications from the individual’s perspective | Qualitative interviews | A (C struggle to stay well, frequent relapses M - fear of relapse, O continue to take medication) | B moderate relevance | A taken from findings |
| Younas,  2016 | UK | PC | To explore the views and experiences of UK mental health pharmacists regarding the use of SDM in antipsychotic prescribing in people diagnosed with SMI. | Qualitative Interviews | C medication working M reluctance to make changes, scary | B relevant but not GP specific | A taken from findings |
