# Supplementary material for: The role of trust and hope in antipsychotic medication reviews between GPs and service users a realist review
Source: BMC Psychiatry. 2021 Aug 4;21:390. doi: 10.1186/s12888-021-03355-3 (PMC8340528; doi:10.1186/s12888-021-03355-3)
Supplement: Supplementary file 4 — Additional file 4. List of included papers. [file 12888_2021_3355_MOESM4_ESM.docx]

### 4. Additional File: List of included papers

Table: List of included papers

| First author | Title | Country | Setting* | Aim | Study Design and data collection |
| --- | --- | --- | --- | --- | --- |
| Adams,  2007 | Shared Decision-Making Preferences Of People With Severe Mental Illness | USA | G | Perceived roles and preferences were explored for shared decision making among persons with severe mental illnesses. | Questionnaire |
| Aref-Adib, 2016 | A Qualitative Study Of Online Mental Health Information Seeking Behaviour By Those With Psychosis | UK | G | To explores the nature, extent and consequences of online mental health information seeking behaviour by people with psychosis and to investigate the acceptability of a mobile mental health application (app). | Qualitative interviews |
| BMJ News, 1995 | Mental Health Law Obsolete, Says Inquiry. | UK | G | news report | News report |
| Boardman, 2008 | Accessing Health Care Professionals About Antipsychotic Medication Related Concerns | Australia | G | To describe SUs’ access to and satisfaction with health care professionals, including nurses, as related to users’ antipsychotic medication concerns. | Questionnaire |
| Britten, 2010 | Resisting Psychotropic Medicines: A Synthesis Of Qualitative Studies Of Medicine - Taking | UK | G | Describe lay perspectives on prescribed psychotropic medicines. | Systematic review of qualitative studies |
| Burns, 1997 | The Primary Care Of Patients With Schizophrenia: A Search For Good Practice | UK | PC | To develop practice for establishing a register and organizing regular reviews; comprehensive assessments; information and advice for patients and carers; indications for involving specialist services; and crisis management. | Consensus group developed good practice guidelines based on current literature |
| Carr, 2004 | Attitudes And Roles Of General Practitioners In The Treatment Of Schizophrenia Compared With Community Mental Health Staff And Patients | Australia | PC | To examines the attitudes and roles of Australian GPs in the treatment of schizophrenia and their relationships with specialist services. | Questionnaires (completed by GPs, mental health staff and service users) |
| Carrick, 2004 | The Quest For Well-Being: A Qualitative Study Of The Experience Of Taking Antipsychotic Medication | UK | G | To outline the experience of taking antipsychotic medication | Qualitative interviews + focus group |
| Corrigan, 2000 | Mental Health Stigma as Social Attribution: Implications for Research Methods and Attitude Change | USA | n/a | To illustrate how attribution model advances research questions related to mental health stigma | Non- systematic literature review |
| Corrigan, 2013 | Erasing the Stigma; Where Science Meets Advocacy | USA | n/a | Review of existing research regarding public stigma reduction, looking at approaches within mental health and other stigmatised communities. | Non- systematic literature review |
| Crawford, 2014 | Assessment And Treatment Of Physical Health Problems Among People With Schizophrenia: National Cross-Sectional Study | UK | G | To examine the quality of assessment and treatment of physical health problems in people with schizophrenia. | Audit of routine data + questionnaire |
| Delman, 2015 | Facilitators And Barriers To The Active Participation Of Clients With SMI In Medication Decision Making: The Perceptions Of Young Adult Clients | USA | G | To explore factors influencing active participation of young SU in psychotropic medication decision making | Qualitative interviews |
| Dixon, 2008 | Medical Students’ Attitudes To Psychiatric Illness In Primary Care | UK | PC | We describe a study of the attitudes and predicted behaviours of medical students towards patients with mental illness in primary care. To investigate the effects that level of undergraduate medical training and personal characteristics might have on responses. | Vignettes (either schizophrenia, depression, diabetes or no illness) and questionnaire |
| Donlon,  1987 | "The Schizophrenias:  Medical Diagnosis And Treatment By The Family Physician" | USA | PC | Overview of care of schizophrenia in primary care | Non – systematic literature review |
| Feeney, 2006 | Atypical Antipsychotic Monitoring: A Survey Of Patient Knowledge And Experience | Ireland | G | To examine the knowledge and experiences of side-effects and their monitoring in patients prescribed atypical antipsychotic medications. | Questionnaire |
| Galon, 2012 | Engagement In Primary Care Treatment By Persons With Severe And Persistent Mental Illness | USA | PC | To describe the social process of engagement in primary care treatment from the perspective of persons with SPMI. | Qualitative interviews |
| Happell, 2004 | Wanting To Be Heard: Mental Health Consumers’ Experiences Of Information About Medication | Australia | G | To examine the experiences of consumers, specifically in relation to education and decision making with regards to medication. | Focus group |
| Hustig, 1998 | Managing Schizophrenia In The Community | Australia | PC | Overview of care of schizophrenia in primary care | MJA Practice Essentials (non systematic literature review) |
| Johnson, 1997 | Professional Attitudes In The UK Towards Neuroleptic Maintenance Therapy In Schizophrenia | UK | G | To assess length of time considered suitable for treatment of schizophrenia | Teleconference between consultant psychiatrists, GPs, pharmacists and CPNs + Questionnaire + commentary |
| Jones, 1987 | Educating Family Physicians To Care For The Chronically Mentally Ill | USA | PC | overview of care of schizophrenia in primary care | Non – systematic literature review |
| Jones, 2015 | Schizophrenia In A Primary Care Setting | UK (but studies from all over) | PC | overview of care of schizophrenia in primary care | Non – systematic literature review |
| Katschnig, 2018 | Psychiatry's Contribution To The Public Stereotype Of Schizophrenia: Historical Considerations | Austria | G | To discuss the origins of the idea of a chronic brain disease, of the split personality concept derived from the term “schizophrenia” , and the craziness idea reflected in the “first rank symptoms”, which are all hallucinations and delusions . | Non – systematic literature review |
| Kendrick, 1995 | Randomised Controlled Trial Of Teaching General Practitioners To Carry Out Structured Assessments Of Their Long Term Mentally Ill Patients | UK | PC | To assess the impact of teaching general practitioners to carry out structured assessments of their long term mentally ill patients. | RCT of structured assessments vs TAU |
| Lambert, 2009 | Are The Cardiometabolic Complications Of Schizophrenia Still Neglected? Barriers To Care | USA mostly | PC | barriers of physical health testing in primary care | Non systematic literature review |
| Lawrie, 1998 | General Practitioners’ Attitudes To Psychiatric And Medical Illness | UK | PC | To examine the attitudes of general practitioners to patients with diﬀerent psychiatric and medical illnesses. | Vignettes |
| LeGeyt, 2016 | Personal Accounts Of Discontinuing Neuroleptic Medication For Psychosis | UK | G | To explore personal accounts of making choices about taking medication prescribed for the treatment of psychosis (neuroleptics). | Qualitative Interviews |
| Lester, 2003 | Satisfaction With Primary Care: The Perspectives Of People With Schizophrenia | UK | PC | This study aimed to explore the elements of satisfaction with primary care for people with schizophrenia. | Qualitative interviews |
| Lester, 2005 | Patients’ And Health Professionals’ Views On Primary Care For People With Serious Mental Illness: Focus Group Study | UK | PC | To explore the experience of providing and receiving primary care from the perspectives of primary care health professionals and patients with SMI respectively | Focus group |
| Magliano, 2017 | Effects Of The Diagnostic Label ‘Schizophrenia’, Actively Used Or Passively Accepted, On General Practitioners’ Views Of This Disorder | Italy | PC | To investigate GPs’ views of schizophrenia and whether they were influenced by a ‘schizophrenia’ label, passively accepted or actively used. | Vignette + Questionnaire |
| Maidment, 2011 | An Exploratory Study Of The Role Of Trust In Medication Management Within Mental Health Services | UK | SC | To develop understandings of the nature and inﬂuence of trust in the safe management of medication within mental health services | Focus groups |
| McDonell, 2011 | Barriers To Metabolic Care For Adults With Serious Mental Illness: Provider Perspectives | USA | PC | This study assessed barriers to metabolic care for persons with serious mental illness (SMI) by surveying experienced healthcare providers. | Questionnaire |
| Mitchel & Selmes, 2007 | Why Don’t Patients Take Their Medicine? Reasons And Solutions In Psychiatry | UK | G | To discuss patients’ reasons for failure to concord with medical advice, and predictors of and solutions to the problem of nonadherence. | Non – systematic literature review |
| Morant, 2016 | Shared Decision Making For Psychiatric Medication Management: Beyond The Micro-Social | UK | G | This conceptual review argues that several aspects of mental health care that diﬀer from other health-care contexts may impact on processes and possibilities for SDM. | Conceptual review |
| Morrison, 2015 | Living With Antipsychotic Medication Side-Effects: The Experience Of Australian Mental Health Consumers | Australia | G | The present study explores people’s experience of living with antipsychotic medication side-effects | Qualitative interview |
| Mortimer  2004 | Atypical Antipsychotics As First-Line Treatments For Schizophrenia  Advantages For Stakeholders In The UK Healthcare System | UK | G | Review on antipsychotic prescribing | Non – systematic literature review |
| Mortimer  2005 | Primary Care Use Of Antipsychotic Drugs: An Audit And Intervention Study | UK | PC | To audit and intervene in the suboptimal prescribing of antipsychotic drugs to primary care patients. | Audit + intervention study |
| NICE, 2014 | PSYCHOSIS And Schizophrenia In Adults | UK | G | Guidelines on treatment and management | Evidence based guideline |
| Oud, 2009 | Care For Patients With Severe Mental Illness: The General Practitioner's Role Perspective | UK | PC | Responsibility and nature of care for people with SMI was explored from a GP perspective | Questionnaire |
| Pereira, 1997 | A Survey Of The Attitudes Of Chronic Psychiatric Patients Living In The Community Toward Their Medication | UK | G | To assess the acceptability of depot among those patients receiving medication via this route and, finally, to assess the views of subjects receiving oral medication about depot. | Questionnaire |
| Pilgrim, 1993 | Mental Health Service Users’ Views Of Medical Practitioners | UK | PC | positive and negative views about general practitioners (GPs) and psychiatrists are examined. | Questionnaire |
| Rasmussen  2006 | Improving Practice | UK | PC | Overview of care of people with SMI for GPs | Non – systematic literature review |
| Roe,  2009 | Why And How People Decide To Stop Taking Prescribed Psychiatric Medication: Exploring The Subjective Process Of Choice | Israel | G | The purpose of the present study was to explore why and how people with a serious mental illness (SMI) choose to stop taking prescribed medication | Qualitative interviews |
| Rogers,  2002 | Some National Service Frameworks Are More Equal Than Others: Implementing Clinical Governance For Mental Health In Primary Care Groups And Trusts | UK | PC | To reports on Primary Care Groups (PCGs) and Primary Care Trusts (PCTs) engaged with the Mental Health National Service Framework (NSF) as part of their remit to implement clinical governance. | Multiple case study |
| Rogers,  1998 | The Meaning And Management Of Neuroleptic Medication: A Study Of Patients With A Diagnosis Of Schizophrenia | UK | G | To describe the meaning and management of neuroleptic medication by people who have received a diagnosis of schizophrenia. | Qualitative interviews |
| Royal College of Psychiatrists | Mental Illness: Stigmatisation And Discrimination Within The Medical  Profession | UK | SC | Report to combat and reduce stigmatisation of people with mental disorders. | Non – systematic literature review |
| Salomon,  2013 | “All Roads Lead To Medication?” Qualitative Responses From An Australian First-Person Survey Of Antipsychotic Discontinuation | Australia | G | The purpose of the survey was to better understand the experiences of people who attempt antipsychotic discontinuation. | Questionnaire |
| Schachter  1999 | Documenting Informed Consent For Antipsychotic Medication  What Family Physicians Should Know | Canada | PC | To educate about informed consent | Editorial |
| Schizophrenia Commission, 2012 | The abandoned illness: a report from the Schizophrenia Commission | UK | G | To examine the provision of  care for people living with psychotic illness. | Non-systematic literature review + survey + visits to services |
| Schulze,  2017 | Stigma And Mental Health Professionals: A Review  Of The Evidence On An Intricate Relationship | Switzerland | SC | To explore ways in which mental health professionals are  ‘entangled’ in anti-stigma activities. It will outline the complex relationships between stigma and the psychiatric profession,  presenting evidence on how its members can be stigmatizers, stigma recipients and powerful agents of de-stigmatization. | Non – systematic literature review |
| Seale,  2007 | Antipsychotic Medication, Sedation And Mental Clouding: An Observational Study Of Psychiatric Consultations | UK | SC | To explore how discussions about side effects are managed in practice | Observational study + Conversation Analysis |
| Toews,  1996 | Improving The Management Of Patients With Schizophrenia In Primary Care: Assessing Learning Needs As A First Step | Canada | PC | To assess family physician learning needs related to the care of patients with schizophrenia. | Questionnaire |
| Tranulis,  2011 | Becoming Adherent To Antipsychotics: A Qualitative Study Of Treatment Experienced Schizophrenia Patients | Canada | G | To explore views on illness and medication use and emphasized key turning points, such as periods of nonadherence and illness relapses. | Qualitative interviews |
| Usher,  2001 | Taking Neuroleptic Medications As The Treatment For Schizophrenia: A Phenomenological Study | Australia | G | To explore the experience of taking neuroleptic medications from the individual’s perspective | Qualitative interviews |
| Viron,  2012 | Schizophrenia For Primary Care Providers: How To  Contribute To The Care Of A Vulnerable Patient Population | USA | PC | This review provides primary care providers with a general understanding of the psychiatric and medical issues speciﬁc to patients with schizophrenia and a clinically practical framework for engaging and assessing this vulnerable patient population | Non- systematic literature review |
| Younas,  2016 | Mental Health Pharmacist’s Views On Shared Decision-Making For Antipsychotics In Serious Mental Illness | UK | G | To explore the views and experiences of UK mental health pharmacists regarding the use of SDM in antipsychotic prescribing in people diagnosed with SMI. | Qualitative Interviews |

*PC =primary care, SC= secondary care, G = about care or treatment in general, without specifically looking at service provision in secondary or primary care services, n/a = setting unrelated to mental health
